# Phage therapy in revision arthroplasty: State of the art and application protocols

**DOI:** 10.1186/s42836-025-00355-6

**Published:** 2026-01-13

**Authors:** Julius Michael Wolfgart, Hanno Schenker, Matthias Gatz, Filippo Migliorini, Joerg Eschweiler, Steffen Langwald, Hans-Peter Horz, Albrecht Eisert, Thomas Schwanz, Ulf Krister Hofmann

**Affiliations:** 1 Department of Orthopaedic, Trauma, and Reconstructive Surgery, Division of Arthroplasty and Tumour Surgery, RWTH University Hospital, 52074 Aachen, Germany; 2 Department of Orthopaedic, Trauma, and Reconstructive Surgery, RWTH University Hospital, 52074 Aachen, Germany; 3 Department of Orthopaedic and Trauma Surgery, Academic Hospital of Bolzano (SABES-ASDAA), Teaching Hospital of the Paracelsus Medical University, 39100 Bolzano, Italy; 4 Department for Trauma and Reconstructive Surgery, University Hospital of the Martin Luther University Halle, 06120 Halle (Saale), Germany; 5 Department for Trauma and Reconstructive Surgery, BG Klinikum Bergmannstrsot Halle, 06112 Halle (Saale), Germany; 6 Institute of Medical Microbiology, RWTH University Hospital, 52074 Aachen, Germany; 7 Hospital Pharmacy, RWTH University Hospital, 52074 Aachen, Germany; 8 Institute of Clinical Pharmacology, RWTH University Hospital, 52074 Aachen, Germany; 9 Division of Infection Control and Infectious Diseases, RWTH University Hospital, 52074 Aachen, Germany

**Keywords:** Phage therapy, Periprosthetic joint infection, Arthroplasty, Musculoskeletal medicine

## Abstract

**Introduction:**

Periprosthetic joint infections (PJI) pose significant clinical challenges due to biofilm formation and antibiotic resistance. Standard treatment often involves implant removal and prolonged antibiotic therapy. Novel strategies target intracellular pathogens and biofilm-associated bacteria, including liposomal antibiotics, antimicrobial peptides, and bacteriophage therapy. Bacteriophages offer specificity and minimal disruption to human microbiota but remain experimental in PJI. Combining phages with targeted antibiotics shows promising results in preclinical models, though further research is needed to confirm efficacy in human PJI and optimise delivery methods.

**Objectives:**

This study updates the current evidence on the use of bacteriophages for patients with PJI, proposing guidelines for their clinical application.

**Method:**

PubMed was searched for articles containing phage therapy in revision arthroplasty. No additional filters or time constraints were used. All eligible studies were accessed by hand.

**Results:**

A total of 39 studies (20 clinical, 19 reviews) on phage therapy for PJI were analysed, covering 56 patients. Of those, negative outcomes were only reported in five. Most studies involved elderly patients with periprosthetic infections of the knee or hip and showed high success rates when combined with antibiotics and surgery. Phage therapy was well tolerated, with only mild adverse effects, such as fever and reversible transaminitis, occurring predominantly with intravenous administration. Review articles reveal that despite promising outcomes, challenges remain, including a lack of standardisation, limited clinical data, and regulatory hurdles.

**Conclusion:**

This study highlights the potential of phage therapy for PJI, emphasising its high specificity, ability to target antibiotic-resistant bacteria, and capacity to disrupt biofilms, and provides a guideline for its clinical administration. Clinical adoption, however, remains limited by regulatory barriers, lack of standardised protocols, and insufficient trial data. Key steps for implementation include establishing regulatory frameworks, developing academic–industrial partnerships and reference centres, and identifying indications supported by controlled trials. With these in place, phage therapy could become a promising adjunct in managing periprosthetic joint infections.

Video Abstract

**Supplementary Information:**

The online version contains supplementary material available at 10.1186/s42836-025-00355-6.

## Background

Periprosthetic joint infections (PJI) remain one of the most challenging complications in orthopaedic surgery. It is not only a localized infection but a complex immunological and microbiological phenomenon that often requires a combination of surgical intervention and prolonged antimicrobial therapy [1]. Although the overall incidence is comparatively low, estimated at 0.5–2% of joint arthroplasty procedures [2], the high and continuously rising number of joint replacements worldwide makes PJIs a major burden for patients and health care systems [3]. These infections are associated with prolonged morbidity, high revision rates, and considerable socioeconomic costs averaging over three times higher than those of uncomplicated primary joint arthroplasties [3]. Consequently, there is an urgent need to explore novel therapeutic strategies, such as phage therapy, to improve outcomes.

## Introduction

### Pathogenesis of PJI

There are two main ways of contaminating an operated joint: the first is through perioperative contamination, the second through haematogenous infiltration [4]. At least when the first route of infection is present, it is unlikely that a monobacterial infection is present, even when just one bacterial strain can be demonstrated in cultures [5]. Resistance to antibiotic treatment is an increasing and increasingly pressing challenge [6]. One of the reasons for antibiotic insusceptibility in PJI is biofilm formation on the vital implants [4]. It protects against the effect of antibiotics, often leading to an increase in the minimal inhibitory concentration up to 1,000-fold, to control the infection compared to their planktonic (free-floating) counterpart [7]. It also impairs the immune system’s efficacy and even hampers mechanical debridement.

### Limitations of current therapies and emerging strategies

The standard of care in chronic PJI is still the removal of the implant, insertion of a drug-eluting cement spacer in the defect, and a two-stage reimplantation of a new prosthesis under extended and sensitivity-tested antibiotic coverage. However, even with these extensive measures, treatment failure can occur because some bacteria can persist in protected niches. One important mechanism contributing to persistence is intracellular colonisation. Colonisation of phagocytic and non-phagocytic cells has been particularly described for one of the most common bacteria responsible for PJI—*Staphylococcus aureus* [8–10]. This makes it important for modern anti-infective treatments also to address the intracellular bacteria to successfully and permanently treat an infection. Of note, for already existing antibiotics, this can be achieved by embedding the drug into liposomes [8]. Also, opsonisation of infected cells for the immune system can be performed with bacteria-specific antibodies [9]. Another promising approach, which is still at an in vitro stage, is the use of antimicrobial peptides [11]. To date, resistance development against antimicrobial peptides appears to be unlikely, and these peptides show little cytotoxicity and immunogenicity. Antimicrobial peptides thus appear ideal candidates to treat chronic PJIs. At present, limiting factors seem to be their high cost and delivery issues, such as the possibility of degradation by host proteases [4].

### Bacteriophages as a therapeutic alternative

**Fig. 1:**
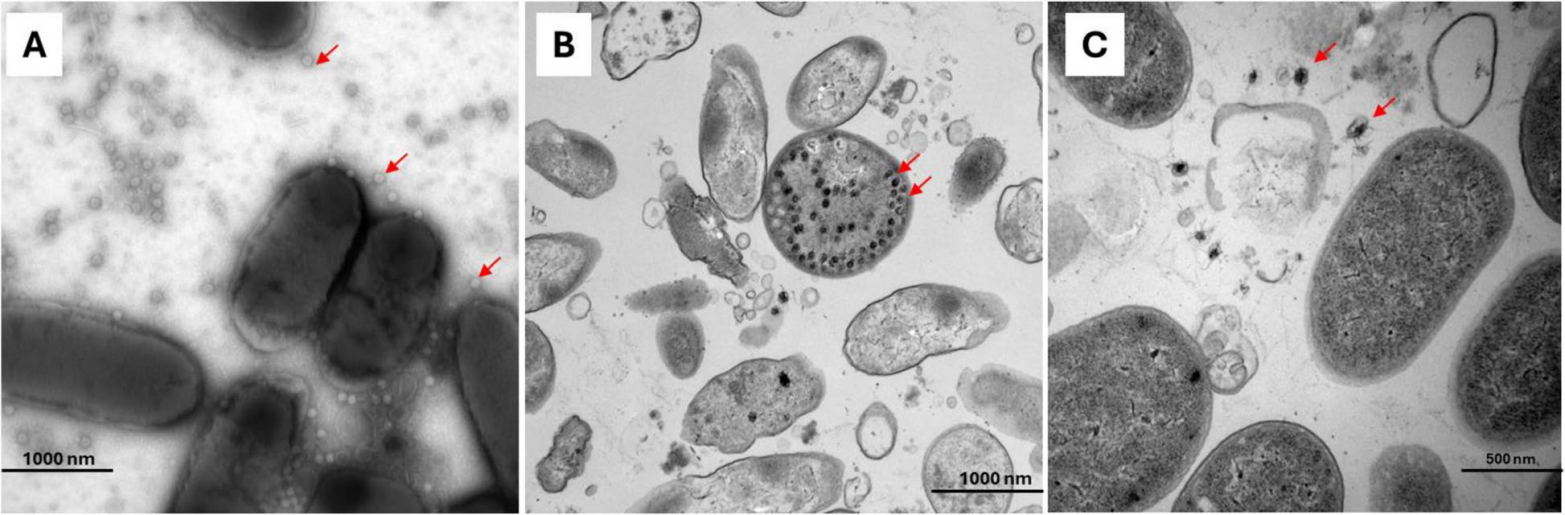
Electron micrograph images showing Escherichia phages adsorbing to the cell surface (**A**), Pseudomonas phages maturating in a soon-to-be-lysed cell (**B**), and Enterobacter phages being released from a lysed cell (**C**). Laboratory samples. *Images were prepared by Eva Miriam Buhl and Hiltrud Königs, Electron Microscopy Facility, RWTH Aachen University Hospital, 52,074 Aachen, Germany*

**Fig. 2:**
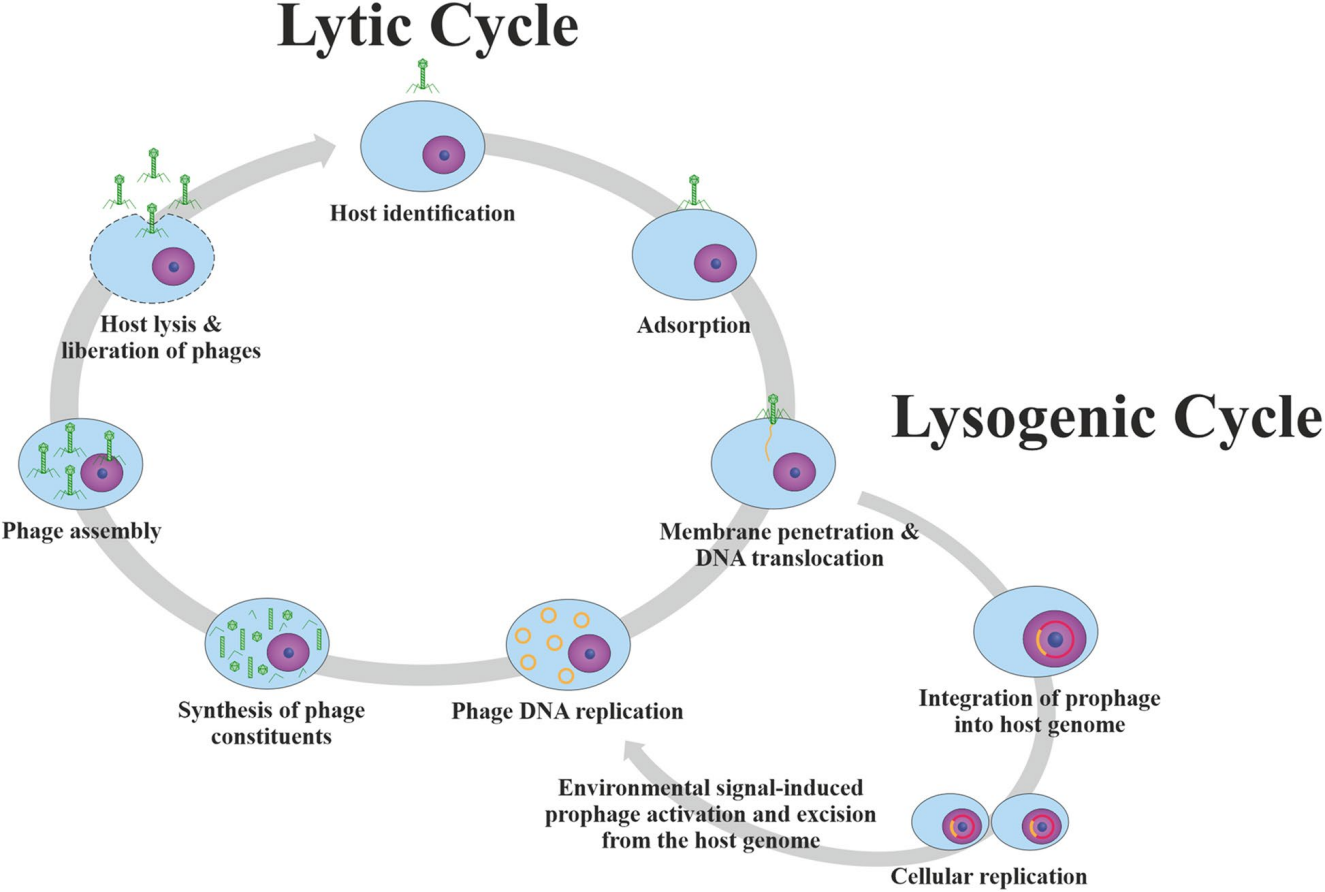
The bacteriophage life cycle involves two principal pathways. *Virulent phages undergo the lytic cycle, during which the host cell is lysed, and progeny virions are released into the surrounding environment. Temperate phages are capable of entering either the lytic or the lysogenic cycle. In certain cases, phages utilize small signaling molecules to coordinate and regulate the decision between lysis and lysogeny. In the lysogenic pathway, the phage genome integrates into the host genome, becoming a prophage. Under specific inducing conditions, the prophage can be activated, resulting in phage gene expression and transition to the lytic cycle*

One treatment strategy gaining increasing scientific interest is the use of bacteriophages (phages), which are viruses that exclusively infect bacteria. In recent years, phages have gained renewed attention as a potential treatment for difficult-to-treat infections. They are considered the most abundant biological particles on earth and are found everywhere where bacteria reside [12]. They possess a high species-specificity towards their target bacteria without affecting the human microbiota [13]. Phages eliminate bacteria by recognising specific surface proteins of the germ and then adsorbing and injecting their genomic material into the bacterium. The bacterium’s replication and translation mechanisms are then repurposed to translate viral mRNA into proteins and to create new phages, which are then released by bacterial lysis and death [13]. Already today, they are in use in the food industry to prevent bacterial contamination (e.g., of *Escherichia coli* and *Salmonella*) in dairy products [14]. In medical use, only such phages are used that exhibit a purely lytic lifecycle (Figs. 1 and 2).

Others, the so-called temperate phages which integrate their genome into the bacterial chromosome, are, so far, not in human use. While the latter can reactivate and enter lytic growth when the host stress response is active (e.g., due to exposure to UV light or a temperature change), they can also provide immunity from further infection from related phages and contribute to horizontal gene transfer that can lead to the spread of antibiotic resistance and toxins [15]. Both categories of phages (i.e., either the obligate lytic or temperate phenotype) can be clearly distinguished based on whole genome sequence analysis, which means that temperate phages can be easily and safely ruled out for therapeutic applications. Although still at an experimental stage in Western countries, scientific works have already been published since the 1960s in the former Eastern Bloc [16]. Especially in the field of bone and joint surgery, phage therapy is still largely unexplored. In a review article, Gibb and Hadjiargyrou reported on 42 clinical trials presently registered on clinicaltrials.gov, none of which, however, addresses periprosthetic joint infections [16].

In animal [17, 18] and in-vitro [19] studies, the administration of phages in conjunction with sensitivity-tested antibiotics appears to obtain by far the best results. The effect of administered phages seems dose-dependent [20]. Phage therapy is not effective against intracellular bacteria in osteoblasts [20], which is one of the reasons why aggressive concomitant antibiotic administration appears integral to the success of the treatment.

### Aim of this review

This article aims to provide an up-to-date overview of the scientific evidence regarding the clinical application of bacteriophages in the treatment of PJI. Special emphasis is placed on their potential role in orthopaedic surgery, where therapeutic alternatives are urgently needed.

## Methods

### Eligibility criteria

All published studies investigating phage therapy in the clinical treatment of periprosthetic joint infections were accessed. Only articles available in English, French, Spanish, and German were eligible. Original studies with evidence of I to IV, according to the Oxford Centre of Evidence-Based Medicine, were considered. Preprints, letters, editorials, corrections, abstracts, and posters were not considered. Animal studies, cadaveric studies, and in vitro studies were not eligible.

### Search strategy

This study was conducted according to the Preferred Reporting Items for Systematic Reviews and Meta-Analyses: the 2020 PRISMA statement [21]. The PICO algorithm was preliminarily established:

- P (Problem): periprosthetic joint infection
- I (Intervention): phage therapy
- C (Comparison): evaluate tool
- O (Outcomes): success of phage therapy in periprosthetic joint infection

On November 17, 2024, the following database was accessed: PubMed. No time constraint was set for the search. Using the building block approach, distinct keywords/blocks were searched (Supplementary File 1). Articles identified through cross-referencing were also added.

### Selection and data collection

Two authors (JMW; UKH) independently performed the database search. All titles were screened by hand, and if suitable, the abstract was accessed. The full text of the abstracts that matched the topic was accessed. A hand cross-reference of the bibliography of the full-text articles was also performed for inclusion. The authors debated and mutually solved disagreements. Only studies on which both investigators fully agreed were subsequently screened. A third senior author (FM) made the final decision in case of further disagreements.

### Data items and outcome of interest

If a study was included, the following information was collected (Table 1): name of authors and year of publication, journal, study design, country of origin, the title and purpose of the article, number of included patients, protocol/design/setup, reported bacteria, and main findings.

**Table 1:** Case reports and clinical studies on phage therapy in revision arthroplasty

Author, Year	Journal Name	Design	Reason for Phage Therapy	Bacterium	Surgery	Phage Therapy Duration	Type of Phage	Dose	Antibiotics	Follow-up (months)	Clinical Result & Main Findings
Ferry et al., 2018 [28]	Open Forum Infectious Diseases	Case Report (*n* = 1)	Periprosthetic joint infection of the right hip	Staphylococcus aureus (and Pseudomonas aeruginosa)	Isolated debridement without replacement of modular parts	1 d	Six phages: among others 1493, 1815, 1957, ns (*n* = 3)	Two intra-articular doses of 10 mL containing three phages (1 × 10^9^ PFU/mL each; total 3 × 10^9^ PFU/mL) were administered	Daptomycin 850 mg was administered intravenously every 24 h for three months, followed by oral amoxicillin 6 g/day plus clindamycin 1.8 g/day for another three months, after which amoxicillin was continued as monotherapy	18	**Clinical Result:** 18 months after the phage therapy injection, the outcome remained favourable, with no clinical signs of persistent infection **Success Rate:** 100% **Main Findings:** The authors report a successful outcome in this case. They state that salvage use of a bacteriophage cocktail was safe and associated with clinical success, as well as potential anti-biofilm activity, in a patient with relapsing Staphylococcus aureus periprosthetic joint infection. Selection of the optimal bacteriophage combination should be guided by phagogram testing of the infecting strain prior to initiating therapy
Patey et al., 2019 [39]	Viruses	Case Series (*n* = 3)	Peripro-sthetic joint infections of the knee (*n* = 2) and hip (*n* = 1)	Staphylococcus spectrum, Staphylococcus aureus, Pseudomonas aeruginosa	Surgery (*n* = 2, not further specified) and no surgery (*n* = 1)	10 days, ns (*n* = 2)	anti-Staphylococcus aureus suspension, commercial broad-spectrum multi-bacteriophage suspension	ns	ns	up to 24	**Clinical Result:** In the case of Pseudomonas aeruginosa, complete bacterial clearance was achieved, although Enterococcus species subsequently emerged. In the case of Staphylococcus aureus infection, a complete cure was observed. In the case of not further specified Staphylococcus species, partial disinfection was achieved, including the closure of several fistulas, followed by clinical stabilization **Success Rate:** 66,7% **Main Findings:** The authors report a successful outcome in at least two of the three demonstrated cases. The authors state that, pending definitive evidence from phage efficacy trials, the compassionate use of phage therapy, combined with appropriate antibiotic treatment, should be continued to optimize outcomes for patients with antibiotic-resistant or otherwise difficult-to-treat infections
Tkhilaishvili et al., 2020 [34]	Antimicrobiological Agents Chemotherapy	Case Report (*n* = 1)	Periprosthetic joint infection of the right knee	Pseudomonas aeruginosa	Three-stage revision	6 days	ns	During stage one surgery, 100 mL of purified bacteriophage was administered, followed by 5 mL (10^8^ PFU/mL) via drains every 8 h for five days	Stage 1: Spacer loaded with gentamicin and clindamycin; colistin 150 mg i.v. every 24 h, meropenem 1 g i.v. every 12 h, ceftazidime 2 g i.v. every 12 h, and daptomycin 700 mg i.v. every 48 h Stage 2: Colistin and ceftazidime were discontinued, and rifampin 600 mg p.o. every 24 h plus doxycycline 100 mg p.o. every 12 h were administered for six weeks	10	**Clinical Result:** At the 10-month follow-up after reimplantation, the patient reported no pain in the right knee. The soft tissue around the surgical site appeared normal, and joint mobility was satisfactory. Serum C-reactive protein levels were within the normal range. Conventional X-ray imaging confirmed proper positioning of the knee prosthesis, with no evidence of implant loosening **Success Rate:** 100% **Main Findings:** The authors report a successful outcome in the demonstrated case. They state that this case indicates a potential adjunctive role of phages for the eradication of multidrug-resistant biofilms with limited therapeutic options
Ferry et al., 2020 [40]	Frontiers in Medicine	Case Report (*n* = 1)	Periprosthetic joint infection of the left knee	Staphylococcus aureus	DAIR with complex soft tissue reconstruction with a free flap	ns	PP1493, PP1815	Seven milliliters of DAC®-Gel containing two phages (1.43 × 10^9^ PFU/mL each; total 2.86 × 10^9^ PFU/mL) were administered intra-articularly	Daptomycin 850 mg i.v. every 24 h and tigecycline 100 mg loading dose followed by 50 mg i.v. every 12 h was administered; after one month, tigecycline was replaced with ceftazidime, ciprofloxacin, and rifampin	12	**Clinical Result:** On postoperative day 5, despite a favourable local appearance of the surgical site, the patient developed a myocardial infarction requiring emergency stenting and initiation of dual antiplatelet therapy. This was soon followed by bleeding at the surgical site, resulting in prosthesis exposure. Consequently, a transfemoral amputation was ultimately performed several months later **Success Rate:** 0% **Main Findings:** The authors state that this case demonstrates the feasibility of using bacteriophage-loaded hydrogel during debridement to treat knee megaprosthesis infection
Ferry et al., 2020 [29]	Frontiers in Medicine	Case Series (*n* = 3)	Periprosthetic joint infection of the left knee	Staphylococcus aureus	DAIR	1 day	PP1493, PP1815, PP1957	Thirty milliliters, reportedly containing three phages (3.3 × 10^8^ PFU/mL each; total 1 × 10^9^ PFU/mL), were administered intra-articularly	Doxycycline (*n* = 2), Cefalexin (*n* = 1)	7 to 30	**Clinical Result:** The treatment resulted in favourable outcomes, with marked clinical improvement in function **Success Rate:** 100% **Main Findings:** The authors report overall successful outcomes across the presented cases. They state that phage therapy holds promise as a salvage option for patients with relapsing Staphylococcus aureus periprosthetic joint infections, particularly when combined with suppressive antibiotic therapy to preserve joint function
Doub et al., 2020 [25]	Antibiotics	Case Report (*n* = 1)	Periprosthetic joint infection of the right knee	Staphylococcus aureus	Three-stage revision	Stage 1: 3 days Stage 2: one application Stage 3: one applica-tion	SaGR51Φ1	Stage 1: Two intraoperative doses of 5.4 × 10^9^ PFU in 10 mL normal saline were administered, followed by daily intravenous doses of 2.7 × 10^9^ PFU in 50 mL for three days (discontinued due to elevated liver enzymes) Stage 2: One intraoperative dose of 2.7 × 10^9^ PFU in 10 mL was administered Stage 3: One intraoperative dose of 2.7 × 10^9^ PFU in 10 mL was administered	Daptomycin 1 g was administered intravenously every 24 h for six weeks	ns	**Clinical Result:** CRP levels normalized within 14 days following the initial surgery (prosthesis removal). No clinical or laboratory signs of infection were observed during the interim spacer exchange procedure. Two months later, a cemented distal femoral megaprosthesis was implanted, with intraoperative cultures yielding negative results **Success Rate:** 100% **Main Findings:** The authors report a successful outcome in this case. Prosthesis salvage was not feasible due to extensive bone erosion. However, the patient’s chronic MRSA periprosthetic joint infection was eradicated using a single virulent bacteriophage, administered intra-articularly and intravenously over three days in the first place, in combination with intravenous antibiotics. After the third intravenous dose of bacteriophage therapy, an unusual, reversible transaminitis prompted the stoppage of bacteriophage therapy, but it was continued later
Ferry et al., 2021 [30]	Frontiers in Medicine	Case Report (*n* = 1)	Periprosthetic joint infection of the left knee	Pseudomonas aeruginosa	Arthroscopic lavage	1 day	PP1450, PP1777, PP1792	Thirty milliliters containing three phages (3.3 × 10^8^ PFU/mL each; total 1 × 10^9^ PFU/mL) were administered intra-articularly	Ceftazidime 6 g/day i.v. Ciprofloxacin 500 mg p.o. every 12 h was administered	12	**Clinical Result:** The patient showed rapid clinical improvement, with resolution of heart failure symptoms and left knee pain. C-reactive protein levels normalized promptly. During the one-year follow-up, the left knee remained clinically normal, with full, pain-free range of motion and ambulation **Success Rate:** 100% **Main Findings:** The authors report a successful outcome in this case. They propose that arthroscopic management, when combined with suppressive antimicrobial therapy, may represent a viable salvage strategy for patients with relapsing Pseudomonas aeruginosa periprosthetic joint infection
Ramirez-Sanchez et al., 2021 [32] (cf. Aslam et al., 2020 [37])	Viruses	Case Report (*n* = 1)	Periprosthetic joint infection of the right knee	Staphylococcus aureus	Two-stage revision	6 weeks	SaGR51ø1	During surgery, 10 mL containing a single phage (2.89 × 10^10^ PFU/mL) was administered intra-articularly, followed by intravenous dosing every 12 h for six weeks (ns). An earlier cycle without surgery, using intra-articular phages at 1.89 × 10^9^ PFU/mL, was unsuccessful	Cefazolin 2 g i.v. every 8 h for six weeks	20	**Clinical Result:** A successful outcome was achieved with phage monotherapy during the second course of phage therapy. The treatment response has proven durable, with no recurrence of methicillin-sensitive Staphylococcus aureus observed 20 months after completion of the second course **Success Rate:** 100% **Main Findings:** The authors report an overall successful outcome in this case after recurrence of infection after a first cycle of bacteriophage therapy. The authors report on the safety and efficacy of both intravenous and intra-articular administration of bacteriophage therapy, the successful use of a single lytic phage, and the development of serum neutralization during prolonged treatment. No adverse events were observed during either course of phage therapy, whether administered intravenously or intra-articularly
Neuts et al., 2021 [31]	Acta Orthopaedica	Case Report (*n* = 1)	Periprosthetic joint infection of the left hip	Enteroco-ccus faecalis	No surgery	Cycle 1: 19 days Cycle 2: 19 days Total: 39 days	Phages, IntestiPhages	Oral suspensions of 10 mL Pyophages in the morning and 10 mL Intestiphages in the evening were administered; exact composition unknown	Cycle 1: Amoxicillin 1 g p.o. every 6 h Cycle 2: Doxycycline 200 mg p.o. every 24 h	36	**Clinical Result:** The patient reported no hip-related symptoms, and follow-up did not warrant repeat culture acquisition **Success Rate:** 100% **Main Findings:** The authors report a successful outcome in the demonstrated case. The authors state that, given the rising rates of antibiotic resistance and the challenge of managing difficult-to-treat periprosthetic joint infections, alternative therapeutic strategies are critically needed. Bacteriophage therapy has emerged as a promising option, particularly due to its capacity to target biofilms commonly associated with periprosthetic joint infections
Cano et al., 2021 [23]	Clinical Infectious Diseases	Case Report (*n* = 1)	Periprosthetic joint infection of the right knee	Klebsiella pneumoniae	No surgery	8 weeks	KpJH46Φ2	Fifty milliliters of normal saline containing a single phage (1.26 × 10^9^ PFU/mL) were administered intravenously every 24 h	Minocycline 100 mg p.o. every 12 h	8.5	**Clinical Result:** Marked clinical improvement was observed in erythema, swelling, pain, range of motion, and functional capacity. Erythema resolved following two doses of phage therapy. Pain scores decreased from 8–9 (scale: 1 = minimal, 10 = severe) to 0. No adverse events were reported, and symptom resolution was sustained post-treatment. Inflammatory markers (ESR, CRP) and proinflammatory cytokines, including IL-6, IFN-γ, and TGF-α, declined over the course of therapy. A slight increase in TNF-α levels was noted **Success Rate:** 100% **Main Findings:** The authors report a successful outcome in the presented case. Phage therapy led to the resolution of local signs and symptoms of infection, along with restoration of function. No treatment-related adverse effects were observed, and the patient remained asymptomatic 34 weeks after completion of phage therapy while continuing minocycline treatment
Doub et al., 2021 [26]	Pharmaceuticals	Case Report (*n* = 1)	Periprosthetic joint infection of the left knee	Staphylococcus epidermidis	DAIR	1 day	PM448	During surgery, 10 mL containing a single phage (2 × 10^9^ PFU/mL) was administered intra-articularly	Ertapenem 1 g i.v. every 24 h for six weeks, and daptomycin 500 mg i.v. every 24 h for six weeks	5	**Clinical Result:** Five months after DAIR and adjunctive bacteriophage therapy, the patient exhibited full knee range of motion with no clinical evidence of periprosthetic joint infection recurrence **Success Rate:** 100% **Main Findings:** The authors report a successful outcome in this case. On the first postoperative day, transient elevations in AST and ALT were observed. As a result, the patient declined the initially planned four-day course of intravenous phage therapy. Liver enzyme levels returned to baseline within two days post-surgery. Notably, the patient’s longstanding aplastic anemia, present for over two years, also resolved following phage therapy
Doub et al., 2022 [36]	Antibiotics	Case Report (*n* = 1)	Periprosthetic joint infection of the right knee	Staphylococcus aureus	Two-stage revision	4 days	Mallokai	During stage one surgery, 10 mL normal saline containing a single phage (1 × 10^9^ PFU/mL) was administered intra-articularly, followed by intravenous infusions of 50 mL (2 × 10^8^ PFU/mL) daily for three days	Stage 1: Ceftaroline 600 mg i.v. every 12 h for six weeks Stage 2: Ceftriaxone 2 g i.v. daily for four weeks, followed by cephalexin 500 mg p.o. every 12 h	12	**Clinical Result:** Twelve months post-treatment, there were no signs of infection recurrence **Success Rate:** 100% **Main Findings:** The authors report a successful outcome in the demonstrated case. They state that this case adds to the growing data supporting the potential use of bacteriophage therapy as an adjuvant to surgical interventions in prosthetic joint infections treatment. Reversible transaminitis was observed after the third day of bacteriophage i.v. administration
Doub et al., 2022 [41]	Acta Orthopaedica	Case Report (*n* = 1)	Periprostatic infection of the left shoulder	Klebsiella pneumoniae	DAIR	6 days	KPI KPII	Postoperatively, 10 mL normal saline containing phage KPI (1 × 10^9^ PFU/mL) were administered via joint drainage for two days, followed by KPII (1 × 10^9^ PFU/mL) for another two days, and then a combination of KPI and KPII (2 × 10^8^ PFU/mL each; total 4 × 10^8^ PFU/mL) intravenously for two days	Ertapenem i.v. every 24 h for six weeks, followed by lifelong amoxicillin–clavulanate suppression therapy	14	**Clinical Result:** At six months post-surgery, arthrocentesis yielded Klebsiella pneumoniae. However, 14 months after receiving bacteriophage therapy, the patient remains free of recurrent periprosthetic joint infection, reports reduced pain, and demonstrates sufficient shoulder range of motion to perform desired activities **Success Rate:** 100% **Main Findings:** The authors report an overall successful outcome in this case. They state that bacteriophage therapy may be a promising adjuvant therapeutic with surgical interventions in the treatment of recalcitrant periprosthetic joint infections to reduce morbidity and mortality
Schoeffel et al., 2022 [33]	Pharmaceuticals	Case Report (*n* = 1)	Peripro-sthetic joint infection of the right knee and right hip	Staphylo-coccus aureus	Four-stage revision	Stage 1: 4 days Stage 2: 1 day	SaWIQ0488ø1	Stage 1: During surgery, 10 mL normal saline containing 1.2 × 10^8^ PFU/mL was administered to the hip and knee joints, followed by intravenous infusions of 50 mL (2.4 × 10^7^ PFU/mL) every 24 h for three days Stage 2: The same intraoperative joint administration (10 mL, 1.2 × 10^8^ PFU/mL) was repeated	Stage 1: Daptomycin i.v. for three weeks, followed by Bactrim DS for three weeks	11	**Clinical Result:** Eleven months after receiving the initial doses of phage therapy, there has been no evidence of recurrence. The patient is now ambulating without the use of a cane, can climb stairs, and can drive using her right leg **Success Rate:** 100% **Main Findings:** The authors report a successful outcome in this case. They state that bacteriophage therapy represents a promising treatment option for prosthetic joint infections. This case further supports its potential, particularly in complex cases where conventional surgical and medical interventions have failed
Cesta et al., 2023 [24]	Open Forum Infectious Diseases	Case Report (*n* = 1)	Periprosthetic joint infection of the right hip	Pseudomonas aeruginosa	DAIR	15 days	Pa53	On day 1, 10 mL (10^8^ PFU/mL) were administered every 8 h via joint drainage, followed by 5 mL (10^8^ PFU/mL) every 8 h thereafter	Meropenem 2 g i.v. every 12 h for two weeks, followed by daptomycin 500 mg i.v. every 24 h for four weeks	24	**Clinical Result:** C-reactive protein levels gradually declined, reaching persistently low values. Clinical examinations revealed no pain, signs of infection, or local inflammation. Whole-body 99mTc-labeled white blood cell scintigraphy demonstrated no pathological uptake, confirming the absence of active infection. At the final follow-up, conducted two years post-treatment, there were no clinical signs suggestive of infection relapse **Success Rate:** 100% **Main Findings:** The authors report a successful outcome in the presented case. No serious adverse events were reported. Personalized bacteriophage therapy, administered alongside meropenem, was well tolerated and demonstrated efficacy in eradicating P. aeruginosa infection
Doub et al., 2023 [27]	Clinical Infectious Diseases	Case Series (*n* = 10)	Peri-prosthetic joint infection of the hip, knee, or shoulder	Enterococ-cus faecalis (*n* = 1), Klebsiella pneumoniae (*n* = 1), Staphylo-coccus epidermidis (*n* = 1), Staphylo-coccus lugdunensis (*n* = 2), Staphylo-coccus aureus (*n* = 5)	DAIR, *n* = 6; One/Two-stage revision, *n* = 4	1 to 5 days	EF-1, KP1&2, PM448, Mallokai, SaWIQ0488Φ1, SaGR51Φ1	Intraoperatively, 20 mL normal saline containing a single phage (5 × 10^8^ PFU/mL) was administered, followed by daily intravenous infusions of 50 mL (2 × 10^8^ PFU/mL) for five days. In five cases, 20 mL (5 × 10^8^ PFU/mL) was also delivered via joint drainage for four days. Some protocols were discontinued early due to elevated liver enzymes	Standard of care antibiotic therapy, not further specified	5 to 30	**Clinical Result:** No patient experienced recurrence of periprosthetic joint infection with the original causative organism. One patient required amputation due to severe soft tissue contractures from rheumatoid arthritis, which impaired wound healing and led to prosthesis exposure. Importantly, at the time of amputation, there was no pathological evidence of osteomyelitis, and intraoperative cultures were negative for microbial growth **Success Rate:** 100% **Main Findings:** The authors report overall successful outcomes across the presented cases. This case series supports the safety and efficacy of adjunctive bacteriophage therapy in the surgical and medical management of periprosthetic joint infections. Reversible transaminitis was observed in five patients, leading to early discontinuation of therapy in some cases. Liver enzyme levels normalized following cessation of phage treatment, and no instances of permanent hepatic injury were reported
Fedorov et al., 2023 [22]	Viruses	Prospective (*n* = 23)	Periprosthetic joint infection of the hip	Staphylococcus epidermidis and Staphylococcus aureus	One-stage revision	≥ 10 d	H143, H178, H182, H184	Six milliliters of an unspecified bacteriophage solution were incorporated into the bone cement during mixing, and 20 mL of a single phage (≥ 1 × 10^5^ PFU/mL) was administered intra-articularly for ten consecutive days	Various antibiotic regimens were used according to the identified pathogens, including cefazolin, vancomycin, daptomycin, ciprofloxacin, and rifampin. After discharge, all patients received either ciprofloxacin 500 mg p.o. every 12 h for 3–5 weeks or rifampin 300 mg p.o. daily for three months	12	**Clinical Result:** Only one patient failed because of a change of the main pathogen to Proteus mirabilis. In the phage group, CRP levels showed a nearly threefold increase by day 3 ± 1 postoperatively (median 86.8 mg/L), followed by a significant 1.7-fold decrease to 17.2 mg/L by day 12 ± 2 (*P* = 0.023). The CRP decline in the phage group was more pronounced and statistically significant compared to baseline, indicating a rapid postoperative inflammatory resolution **Success Rate:** 95,7% **Main Findings:** The authors report overall successful outcomes across the presented cases. The relapse rate of periprosthetic joint infection was significantly lower in the phage-treated group compared to the control group receiving only one-stage revision and antibiotics (4.5% vs. 36.4%; *P* = 0.021). No adverse events were reported, apart from transient fever in two patients following phage administration
Onallah et al., 2023 [38]	Open Forum Infectious Diseases	Case Series (*n* = 3)	Periprosthetic joint infections	Enterococ-cus faecalis, Staphylo-coccus aureus	ns	ns	EFGrNG, EFGrKN, SaWIQ493Ph1	ns	Ampicillin, Ceftriaxone, Cefazoline	3 to 12	**Clinical Result:** Ultimately, remission was achieved in two cases, while treatment failure was observed in one. Notably, one patient was presented as two cases. The initial phage therapy cycle was limited in duration due to an insufficient supply of phage preparation for intravenous administration, which was suspected to contribute to the clinical failure and recurrence within one year. However, a second, more adequately administered cycle of phage therapy subsequently led to sustained clinical remission lasting at least one year **Success Rate:** 66,7% **Main Findings:** The authors ultimately report successful outcomes in the demonstrated cases. They state that the use of phages with additional therapy resulted in a high response rate
Doub et al., 2023 [35]	IDCases	Case Report (*n* = 1)	Periprosthetic joint infection of the left knee	Enterococci faecalis	No surgery	6 days	EF phage 1	Intra-articular arthrocentesis was performed for two consecutive days with 10 mL of a single phage (1 × 10^10^ PFU/mL; total 1 × 10^9^ PFU), followed by intravenous administration of 50 mL of the same phage (1 × 10^10^ PFU/mL; total 2 × 10^8^ PFU) daily for four days	Daptomycin 1 g i.v. every 24 h for seven days, followed by amoxicillin 500 mg p.o. every 12 h	24	**Clinical Result:** Twenty-four months after receiving bacteriophage therapy, the patient shows no clinical signs of recurrent periprosthetic joint infection in the left knee joint. A PET/CT scan performed 20 months after therapy revealed no increased uptake around the left knee prosthesis **Success Rate:** 100% **Main Findings:** The authors report a successful outcome in the demonstrated case. They state that bacteriophage therapy for prosthetic joint infections has promise to reduce the morbidity associated with current treatments

## Results

**Fig. 3:**
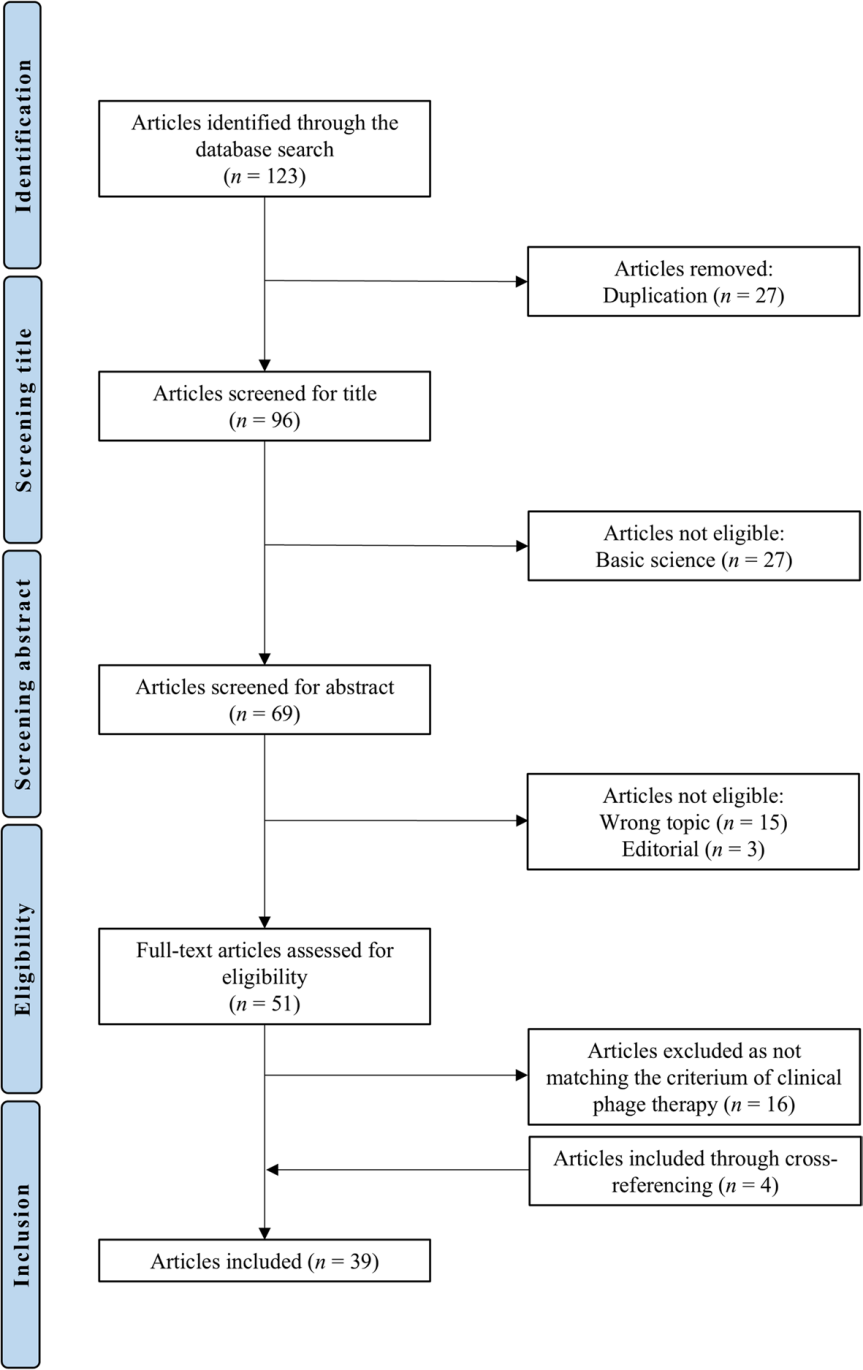
PRISMA flow chart of the literature search

The literature search resulted in a total of 123 articles; after removing duplicates, 96 articles remained. Following the screening process, 39 articles met all predefined inclusion criteria (Fig. 3, Table 1, Table 2).

**Table 2:** Review articles on phage therapy in revision arthroplasty

Authors	Journal	Country of origin	Purpose and key statements
Gemmel et al., 2012 [60]	European Journal of Nuclear Medicine and Molecular Imaging	Belgium	This review provides an insight into radioactively labelled phages for imaging. Early investigations with radiolabelled phages demonstrate limited host specificity and low success rates; although targeted phages may ultimately facilitate combined diagnosis and therapy, their application in PJI remains experimental pending further refinement and clinical validation
Tzeng et al., 2014 [57]	Diagnostic Microbiology and Infectious Disease	USA	This review primarily deals with PJI, including one paragraph about phage therapy. The authors propose exploring phage therapy as a biofilm-targeted treatment for PJI, noting its age-independent activity, extracellular matrix degradation, and site-specific replication, and recommend its use alongside antibiotics to enhance efficacy and limit resistance, contingent on precise pathogen identification
Gbejuade et al., 2014 [58]	Acta Orthopaedica	UK	This review primarily deals with biofilms in PJI, including one paragraph on phage therapy. The authors note that bacteriophages, used as alternatives or adjuncts to antibiotics for biofilm-related infections, may enhance bacterial clearance when combined with antibiotics, but their narrow spectrum and potential immunogenicity necessitate further investigation of safety, efficacy, and administration routes before clinical application
Taha et al., 2018 [4]	Current Reviews in Musculoskeletal Medicine	Canada, USA	This review primarily deals with PJI, including one paragraph about phage therapy. The authors describe phage therapy as a targeted approach that selectively lyses bacteria, including resistant strains, and disrupts biofilms—particularly in combination with antibiotics to enhance killing and limit resistance—yet, despite promising case reports, it lacks regulatory approval and requires further validation, especially in relevant animal models of periprosthetic joint infection
Akanda et al., 2018 [43]	Journal of Orthopaedic Research	Canada	This review primarily deals with phage therapy in PJIs. In addition, it summarizes key findings from animal studies in this regard. The authors identify phage therapy as a promising adjunct for PJI, capable of disrupting biofilms and eradicating bacteria, including persister cells, with enhanced efficacy when combined with antibiotics, but emphasize that despite encouraging preclinical data and the use of strain-specific phage cocktails, further research is required to establish optimal dosing, delivery, and integration into future treatment strategies
Van Belleghem et al., 2020 [44]	Frontiers in Microbiology	USA	This review primarily deals with phage therapy in PJIs and gives an overview of common bacterial pathogens and corresponding available phages. The authors advocate considering phage therapy as a novel approach for PJI, highlighting its ability to degrade biofilms and enhance antibiotic efficacy, its potential integration into surgical and antimicrobial protocols with pathogen-specific targeting, and the use of hydrogels or surface coatings for sustained release, while emphasizing the need for further studies to reduce reliance on invasive surgery and combat antibiotic resistance
Onsea et al., 2020 [45]	European Cells & Materials	Belgium	This review primarily deals with phage therapy in PJIs. The article summarizes key findings from animal studies and provides an overview of 11 studies on phage therapy for musculoskeletal infections, including three cases of PJI. The authors highlight phage therapy as a promising alternative for PJIs unresponsive to antibiotics, emphasizing the need for specific lytic phages, stable local delivery systems, and exploitation of phage–antibiotic synergy, while noting that overcoming regulatory barriers and developing standardized protocols could expand treatment of multidrug-resistant infections, pending further investigation into immune interactions, pharmacokinetics, and resistance patterns
Ferry et al., 2020 [46]	SICOT-J	France	This review primarily deals with phage therapy in PJIs while focusing on medicolegal issues. The authors emphasize that phage therapy should be applied within strict legal and scientific frameworks, with compassionate use limited to cases lacking alternatives, production compliant with Good Manufacturing Practice, administration approved through multidisciplinary consensus, and clinical trials conducted transparently to assess safety and efficacy, supported by robust data-sharing and rigorous regulation of funding, access, and follow-up by national health systems
Doub et al., 2021 [47]	Open Forum Infectious Diseases	USA	This review primarily deals with phage therapy in PJIs. The authors suggest exploring phage therapy as an adjunct for chronic prosthetic joint infections, noting that when combined with DAIR it may eradicate infection without implant removal, provided strain-matched phages with proven in vitro activity are used and delivered intraoperatively and via short-term intravenous administration, while acknowledging that clinical validation is needed to confirm safety and efficacy and that challenges such as narrow host range and pharmacokinetics persist
Walter et al., 2021 [48]	Der Orthopäde	Germany	This review primarily deals with phage therapy in PJIs, while also including other musculoskeletal infections. Eleven case reports or series involving PJIs are included. The authors report that phage therapy, administered intravenously, locally, or via drainage alongside antibiotics, shows potential for PJI eradication amid rising antibiotic resistance, with current case series indicating safety and possible efficacy, but emphasize the need for standardized protocols, randomized trials, and regulatory frameworks to enable routine clinical use and optimize this alternative therapy against increasingly hard-to-treat pathogens
Ferry et al., 2021 [49]	Viruses	France	This review provides an overview of phage therapy for bone and joint infections, including PJI, and gives a summary of what is needed to implement phage therapy in healthcare systems. The authors describe phage therapy as a promising option for refractory PJI, requiring pharmaceutical-grade lytic phages selected for strain-specific activity and administered intraoperatively with antibiotics and surgery under strict multidisciplinary oversight, in accordance with regulatory frameworks mandating compassionate use approval and quality control, and recommend clinical trials to assess its safety and efficacy given its biofilm-disrupting and antibiotic-synergistic effects
Doub et al., 2022 [50]	Journal of Orthopaedic Research	USA	This review provides a tabular summary of challenges and research needs for bacteriophage therapy in PJI. The authors consider bacteriophage therapy a promising approach for PJI, noting its biofilm-degrading and bacteriolytic potential but emphasizing the need to address limitations by clarifying bacterial clonality, anticipating resistance, studying pharmacokinetics, formulating diverse non-competing phage cocktails, standardizing biofilm-specific lytic activity tests, and investigating phenotypic changes and macromolecular interactions that may impede attachment, to enable reproducible and effective treatment
Khalifa et al., 2023 [51]	Journal of Experimental Orthopaedics	Egypt	This systematic review summarizes all case reports of phage therapy in PJI until March 2023. The authors highlight bacteriophage therapy as a promising adjuvant for resistant PJIs after hip and knee arthroplasty, recommending its use with surgical debridement and suppressive antibiotics when conventional treatments fail or revision surgery is not feasible, and stressing the need to confirm safety, clarify dosing, administration routes, and regulatory guidelines to enable broader clinical integration
Steadman et al., 2023 [55]	Antibiotics	Australia, Germany	This review primarily deals with PJI, including one paragraph about phage therapy. The authors present phage therapy as a promising option for PJI refractory to antibiotics, noting its specificity, biofilm-degrading enzymes, and potential benefits when combined with antibiotics intra-articularly or intravenously, but emphasize that wider adoption depends on creating standardized phage libraries, overcoming regulatory barriers, and generating robust clinical evidence
Jevnikar et al., 2024 [59]	Journal of Clinical Orthopaedics and Trauma	USA	This review primarily deals with PJI, including content about phage therapy. The authors emphasize the importance of considering bacteriophage therapy in PJI management for its targeted, microbiota-sparing activity against resistant biofilm-embedded pathogens, recognizing its potential as a complementary or alternative strategy to antibiotics, particularly in cases of treatment failure or multidrug resistance
Piuzzi et al., 2024 [56]	Journal of Orthopaedic Research	USA	This review primarily deals with PJI, including a few information about phage therapy. The authors stress that phage therapy should be developed as a viable alternative for biofilm-associated infections unresponsive to antibiotics, requiring pathogen-specific phages capable of penetrating biofilms, evaluation of immune responses, and supportive regulatory frameworks to enable its clinical use in periprosthetic joint infections amid rising resistance
Eiselt et al., 2024 [52]	European Journal of Microbiology & Immunology	Germany	This systematic review summarizes all case reports of phage therapy in PJI associated with Staphylococcus aureus until 10th of July 2023. The authors propose phage therapy as an adjunct for *Staphylococcus aureus* prosthetic joint infections, highlighting tailored lytic phages and lysins active against planktonic, biofilm-associated, and methicillin-resistant strains, with preclinical and clinical data showing reduced bacterial burden, biofilm disruption, and functional recovery, but advise liver function monitoring, phagogram-guided selection, strict oversight, and randomized trials to establish efficacy
Ferry et al., 2024 [53]	EFORT open Reviews	France, Belgium	This systematic review summarizes all case reports of phage therapy in musculoskeletal infections and PJI up to approximately 2024 and provides an overview of established clinical programs: PHAGEFORCE, PHAGEinLYON. The authors present phage therapy as a promising option for PJI when prosthesis exchange is not feasible, emphasizing multidisciplinary, sensitivity-guided use in complex or relapsing cases, typically via intra-articular injection during DAIR or under sonographic guidance with concurrent antibiotics, while noting the need for standardized protocols and robust evidence to confirm its role
Yang et al., 2024 [54]	International Orthopaedics	France	This systematic review summarizes all case reports of phage therapy in PJI until May 2024 and provides an overview of clinical programs and application schemes: PHAGEFORCE, PHAGEinLYON, and PhagoDAIR. The authors highlight bacteriophage therapy as a promising adjunct for PJI after hip and knee arthroplasty, reporting high infection clearance rates, rare recurrences, mild adverse effects, and notable biofilm and multidrug-resistant *Staphylococcus aureus* activity, but stress the need for larger randomized controlled trials to confirm efficacy and safety across broader patient populations

**Fig. 4:**
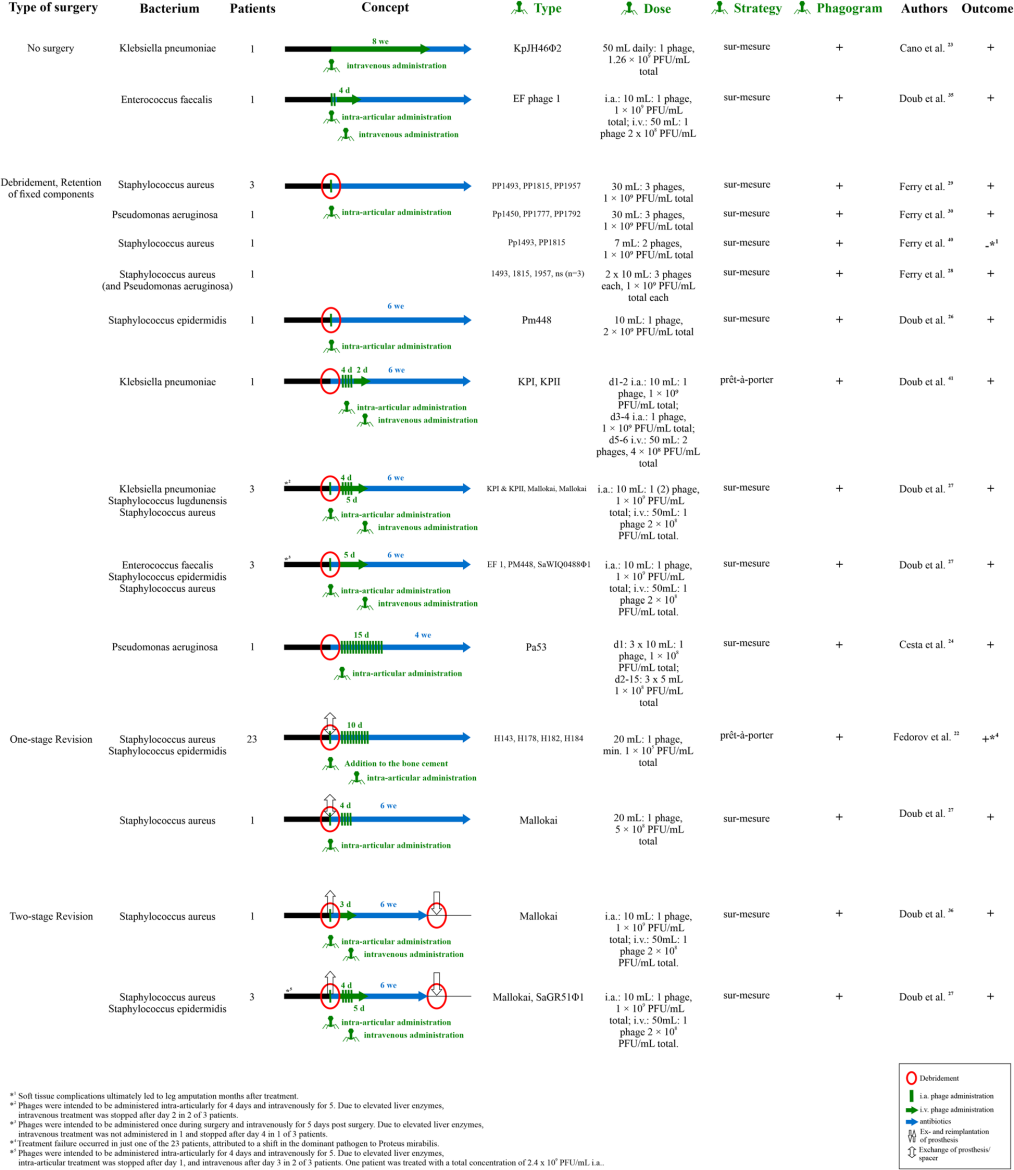
Different treatment strategies applied in the different case reports and case series from the literature. *Columns specifying the type of surgery, bacterial pathogen, number of patients, phage type, phage dose, therapeutic strategy (prêt-à-porter (off-the-shelf) or sur mesure (tailor-made)), pre-sensitivity testing in the form of a phagogram, authors, and clinical outcome corresponding to a distinct concept of phage therapy (rows) in the context of periprosthetic joint infections. Abbreviations: i.a.: intra articular; i.v.: intra venous; d—days; PFU: plaque forming units; we: weeks*

These 39 studies were categorised into two groups: (1) case reports and clinical studies (20 studies) (Table 1), and (2) review articles (19 studies) (Table 2). The search strategy did not include a publication date limit. The first publication on that topic was from 1993. A total of 56 documented cases across various studies were analysed. An overview of the performed treatment approaches is presented in Fig. 4. Most studies presented a case report design, and only one employed a prospective study design by Fedorov et al. [22]. Patients were predominantly elderly, with a median patient age of 70 years, with a standard deviation of 10.41 years across studies. The gender distribution was roughly balanced across the studies. Nineteen out of 20 studies reported successful clinical outcomes of their cases, with no recurrence of infection during the respective follow-up periods [22–39]. Six studies reported an unsuccessful therapy at least in parts of their cases [22, 32, 37–40]. Eight out of 20 studies followed a concept including implant retention [24, 26–30, 40, 41]. Seven studies reported cases in which one-, two-, three, or four-stage revision procedures were performed [22, 25, 27, 32–34, 36]. In just a few cases, surgical intervention was waived [23, 31, 35, 39].

Phage therapy was typically administered over varying durations, from as short as one day [33] to complex regimens spanning several weeks with intermittent pauses [37]. The follow-up ranged from three months [38] up to three years [31]. Most reported bacteria responsible for infection were *Staphylococcus aureus* in 12 of 20 studies [22, 25, 27–29, 32, 33, 36–40]. Followed by *Pseudomonas aeruginosa* [24, 30, 34, 39] and *Enterococcus faecalis* [27, 31, 35, 38]. Others reported *Staphylococcus epidermidis* [22, 26, 27], *Klebsiella pneumoniae* [23, 27, 41], and *Staphylococcus lugdunensis* [27]. Furthermore, microbiological monitoring revealed no major shifts in pathogen profiles following phage application. There were also no clear reports of phage resistance, although this was not systematically investigated across all cases. Relevant adverse events associated with phage therapy were reported. Fever and chills occurred in three cases [22, 24], while transaminitis was observed in nine patients [25–27, 33, 36] with no long-term hepatic impairment. No study reported severe allergic reactions or systemic toxicity. Young et al. [42] conducted a meta-analysis including four available case series by Fedorov et al. [22], Doub et al. [27], Ferry et al. [29] and Onallah et al. [38] with corresponding clinical success rates of 95.7% (22/23 patients), 100% (10/10 patients), 100% (3/3), and 66.7% (2/3 patients). They demonstrated an aggregate proportion of patients achieving clinical remission of their infection of 0.78 (95% confidence interval, CI: 0.39, 0.95) (*I*^2^ = 55%, *P* = 0.08) with evidence of moderate heterogeneity [42]. Another case report by Patey et al. [39] reported a clinical success rate of 66.7% (2/3 patients).

The 19 included review articles (Table 2) primarily focus on PJIs, with many emphasizing phage therapy as a promising adjunctive treatment; several of these reviews also systematically review case reports on the use of phage therapy in PJI. Akanda et al. [43] and Van Belleghem et al. [44] provided early overviews, summarizing findings from relevant animal studies and discussing the range of bacterial pathogens and corresponding therapeutic phages. Onsea et al. [45] extended this work by reviewing 11 studies on musculoskeletal infections, including three PJI cases, while Ferry et al. [46] explored the medicolegal aspects of clinical phage therapy application. Subsequent focused reviews by Doub et al. [47] and Walter et al. [48] emphasized clinical experiences and case series, whereas Ferry et al. [49] outlined the requirements for integrating phage therapy into healthcare systems. A tabular summary of research challenges and needs was later presented by Doub et al. [50]. More recent systematic reviews have consolidated the evidence base: Khalifa et al. [51] summarized all reported PJI phage therapy cases up to March 2023; Eiselt et al. [52] focused specifically on *Staphylococcus aureus* PJIs; Ferry et al. [53] expanded the scope to musculoskeletal infections and discussed established clinical programs such as PHAGEFORCE and PHAGEinLYON. Finally, Yang et al. [54] provided the most up-to-date systematic review (up to May 2024), including recent application schemes like PHAGEFORCE, PHAGEinLYON, and PhagoDAIR. Other authors conducted reviews mainly focusing on PJIs in general, including only single paragraphs on phage therapy [4, 55–59] or special topics like radioactively labelled phages for image-based infection detection [60]. Across review articles, phage therapy is highlighted as a highly specific and targeted approach that lyses pathogenic bacteria without harming human cells or the microbiota. This specificity also allows phages to act effectively against antibiotic-resistant strains and to disrupt biofilms, especially when combined with antibiotics. They advocate for phage-antibiotic combinations, which are believed to enhance bactericidal effects and help prevent the development of resistance. The literature identifies several limitations and challenges to widespread adoption. These include the need for standardised phage libraries, more robust clinical data, and clear regulatory pathways. Animal studies and well-designed clinical trials are recommended to further evaluate safety, efficacy, and clinical benefits, particularly for treating PJIs. Overall, authors report that phage therapy is considered a promising approach in PJI management, but remains in the experimental or compassionate-use stage [4, 43–60].

## Discussion

### Success and failure rates of phage therapy in PJIs

The overall success rate in the documented cases appears remarkably high, with only five failures among 56 patients. The five available case series by Fedorov et al. [22], Doub et al. [27], Ferry et al. [29], Onallah et al. [38], and Patey et al. [39] reported corresponding success rates of 95.7% (22/23 patients), 100% (10/10 patients), 100% (3/3), 66.7% (2/3 patients), and 66.7% (2/3 patients). An ultimately negative outcome was reported in only two patients, and a partially negative outcome in three cases:

1. Fedorov et al. [22] described a failed case of periprosthetic hip infection caused by multi-resistant *Staphylococcus epidermidis*. A one-stage revision surgery was performed, with phages applied both via bone cement and through intra-articular administration for at least ten days, accompanied by antibiotics. The authors attributed the treatment failure to a shift in the infecting pathogen to *Proteus mirabilis*. Considering that both antibiotics and phages exhibit pathogen-specific activity, the therapeutic failure in this case appears plausible and may have been preventable through the substitution of a more appropriate phage, as proposed, e.g., by Aslam et al. [37]. Notably, this was the only unfavourable outcome among the 23 reported cases from this study, reaching an overall success rate of 95.7%, and suggesting that methodological flaws are unlikely to have played a significant role in this one treatment failure. Of note, the incorporation of phages into bone cement requires careful consideration of thermal stability, as the exothermic polymerization of polymethylmethacrylate can reach temperatures that may inactivate phages (starting from 45–50 °C [61]). Standardized protocols to ensure their viability during application are not yet established.
2. Ferry et al. [40] presented one failed case of periprosthetic knee infection caused by multi-resistant *Staphylococcus aureus*. The patient had a megaprosthesis and presented with two fistulas and prosthesis exposure. Initial management included surgical treatment following the established approach, combined with complex soft tissue reconstruction. Phages were administered intraoperatively via DAC®-hydrogel. In the days following surgery, the patient suffered a myocardial infarction, necessitating emergency stenting and initiation of dual antiplatelet therapy. This led to significant bleeding at the surgical site, extensive soft tissue damage, and ultimately, limb amputation several months later. Despite the negative clinical outcome, the treatment failure of phage therapy in this case cannot be conclusively determined.
3. Onallah et al. [38] reported a case of periprosthetic hip infection caused by *Enterococcus faecalis,* which failed in the first instance but was later treated successfully in a second cycle. In this case, neither the type of surgery nor the exact intravenous phage dosage was specified. Concomitant antibiotics were administered. The authors noted that the initial phage therapy cycle was limited in duration due to an insufficient supply of the phage preparation, which may explain the temporary negative outcome, particularly given that the same phage was used in both treatment courses.
4. Ramirez-Sanchez et al. [32] described a similar course of events. This case of periprosthetic knee infection with *Staphylococcus aureus*, also included in a case series by Aslam et al. [37], involved an initial treatment consisting of a single intra-articular knee injection without surgical intervention, followed by intravenous phage administration in combination with antibiotics. Phage therapy was intended for six weeks; however, it was discontinued after two weeks due to supply limitations from the manufacturer. The second, successful treatment course involved a two-stage revision surgery combined with six weeks of intravenous phage therapy—now with the phage concentration increased from 1.89 × 10^9^ to 2.89 × 10^10^—and antibiotics. Although the initial course was cut short, with intravenous administration lasting only two weeks instead of the intended six, and lower phage concentration, this still represents a substantial exposure compared to other therapeutic protocols. While the absence of surgical intervention in the first course may appear limited, it may not always be required, as demonstrated by Doub et al. [35], who reported clinical remission following intra-articular phage administration for two days and intravenous administration for four days, without surgery. Other cases with positive outcomes without surgical intervention were reported by Cano et al. [23], Neuts et al. [31], and Patey et al. [39].
5. Patey et al. [39] also reported a case from their series involving a knee prosthesis infection caused by *Pseudomonas aeruginosa*, which was treated with phages without surgical intervention. Unfortunately, the route of phage administration was not specified. While the therapy led to the clearance of *Pseudomonas aeruginosa*, the appearance of *Enterococcus species* was subsequently observed. Given the limited information, this outcome would be interpreted as negative, resulting in an overall success rate of 66.7% in this case series. Once again, the absence of surgical intervention may appear restrained, but it might have been the only feasible option in this case.

Among all reported cases of phage therapy in PJIs, negative outcomes remain remarkably rare. With only five failures among 56 patients, the observed success rate exceeds 90%. Given that these cases often represent infections refractory to conventional treatment, such results are particularly compelling. Finding patterns in both successful and unsuccessful cases is challenging due to the low number of cases and the highly individual nature of each case. A common factor in two negative—only temporarily in one—outcomes reported by Ramirez-Sanchez et al. [32] and Patey et al. [39] was the omission of surgical intervention, suggesting that the absence of surgery may have contributed to treatment failure. On the other hand, three successful cases where no surgery was performed were reported [23, 31, 35]. However, in four of these five cases where no surgery was performed, it was not intended and was due to the patient’s multimorbidity [23, 31, 35, 39]. The other two surgical concepts that failed in combination with phage and antibiotic therapy were debridement, antibiotics, and implant retention (DAIR) [40] and one-stage revision [22]. But at least 14 (not all the authors specified the type of surgery that was performed) other cases were treated successfully using a DAIR concept, and at least 23 cases were successfully treated using a one-stage revision concept, which suggests that the success rate of these procedures is still high in combination with phages and antibiotics. Of note, all two- or multi-stage revision cases were successful, indicating a potentially superior treatment approach, which in the case of a two-stage revision coincides with gold standard treatment of periprosthetic infections [62].

### Phage administration protocols

Most authors used a total phage concentration of approximately 1 × 10^9^ PFU/mL for preparations administered directly to the local infection site, either intraoperatively or via post-operative intra-articular drainage. The phage solution was usually prepared using normal saline with volumes between ten and 20 mL for intra-articular administration and 50 mL for intravenous administration. Preparations for intravenous application were manufactured with a slightly lower concentration of approximately 2–4 × 10^8^. When phage cocktails containing two or more phages were employed for intra-articular usage, the individual phages were typically present in equal proportions to achieve the total concentration of 1 × 10^9^ PFU/mL. In the case of therapy failure, (1) Fedorov et al. [22] used a concentration of at least 1 × 10^5^ PFU/mL for their phage preparation, while only administering intra-articularly. (2) Ferry et al. [40] used a concentration of 2.86 × 10^9^ PFU/mL in a DAC®-Gel that was administered once during surgery. (3) Unfortunately, Onallah et al. [38] did not provide any information regarding the concentration of phages used. (4) Ramirez-Sanchez et al. [32] used a concentration of 1.89 × 10^8^ PFU/mL for their first therapy cycle, which resulted in recurrence of infection; the same concentration was used for intra-articular and intravenous use. (5) Patey et al. [39] did not include specific information on concentrations as well. Based on all 46 [22, 24–30, 32–36, 41] successful cases with detailed information on phage preparation and duration of local application, a weighted mean concentration of 1.08 × 10^9^ PFU/mL for intra-articular administration can be calculated. Excluding the case series of Fedorov et al. [22] due to inaccurate specifications, the weighted mean concentration increases to 2.08 × 10^9^ PFU/mL. Of these 46 cases, intravenous administration was used in only 14 [25, 27, 32, 33, 35, 36, 41], raising questions about its therapeutic necessity, especially given that transaminitis occurred in 7 of these 14 cases [25–27, 33]. The weighted mean total duration of intra-articular phage therapy in these 46 cases was 6.2 days. The initial dose was typically administered during surgery, with subsequent doses delivered via drainage or a Hickman catheter. Of note, in case of two-, three-, or four-stage revisions, the total duration might extend over multiple stages.

### Proposal of a therapeutic concept

**Fig. 5:**
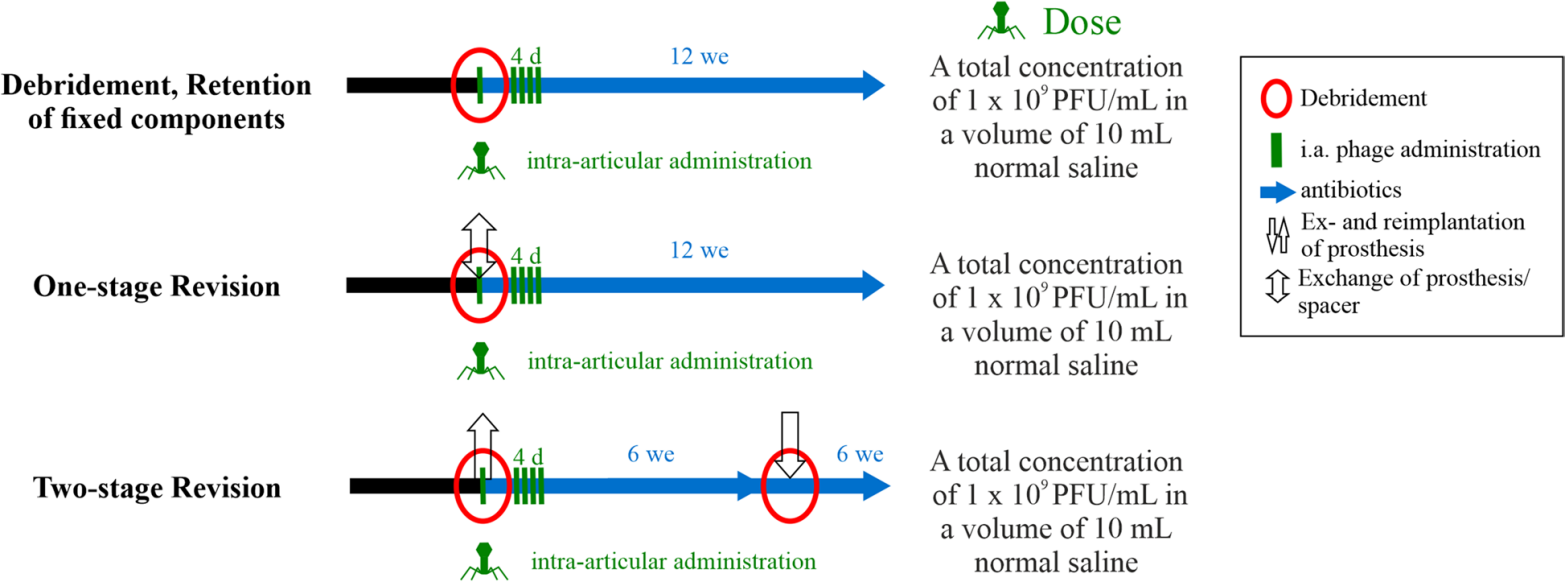
Proposed scheme for phage therapy in combination with Debridement, Antibiotics, and Implant Retention (DAIR), one-stage, and two-stage revision arthroplasty. *In the DAIR approach, phages are administered intra-articularly following mechanical debridement and removal of all exchangeable components. In one-stage revision surgery, phages are delivered intra-articularly after removal of the prosthesis and mechanical debridement. In two-stage revision surgery, phages are administered intra-articularly after prosthesis removal and mechanical debridement, and before implantation of the antibiotic-loaded cement spacer. For all approaches, postoperative intra-articular administration of the phage solution is continued for four consecutive days *via* drainage or a Hickman catheter. Phages may be applied at a total concentration of 1* × *10⁹ PFU/mL in 10 mL of normal saline. Phage therapy should be combined with appropriate antibiotic treatment, for example, as recommended by the PRO-IMPLANT Foundation. Abbreviations: i.a.: intra-articular; d: days; PFU: plaque-forming units; we: weeks*

Combining the literature findings for successful surgical and phage administration strategies we venture proposing one possible concept for recalcitrant late onset PJIs: A two-stage revision protocol including five days of intra-articular phage administration at a total concentration of 1 × 10^9^ PFU/mL in 10 mL normal saline, a regimen also supported by Pirnay et al. [63] based on their experience with nineteen cases of recalcitrant musculoskeletal infections. The first dose is administered intraoperatively during stage one surgery, which includes implant removal, surgical debridement, phage application, and implantation of an antibiotic-loaded cement spacer, while the remaining doses are delivered on the first four post-operative days via drainage or a Hickman catheter (Fig. 5). A single-stage revision may be performed using a similar approach. In selected cases of acute infection following multiple prior conventional two- or one-stage revisions, our findings support the “PhagoDAIR” concept as proposed by Ferry et al. [29]. The surgical approach involves retention of fixed implant components, thorough debridement, and a single intra-articular administration of phages at a concentration of 1 × 10^9^ PFU/mL, delivered in an appropriate volume of normal saline (e.g., 10 mL). The selection of proper phages should be guided by phagogram results and discussed in consultation with the manufacturer. In addition, all approaches must be supported by a tailored antibiotic strategy, for example, as proposed by the PRO-IMPLANT Foundation [64].

The motivation to develop new therapeutic strategies for PJIs arises from the substantial burden these infections place on both the patient and the healthcare system. Ultimately, the increasing number of cases in which treating physicians are unable to identify effective therapies due to the alarming rise in bacterial resistance further underscores the need for alternative treatment approaches. For precisely such cases, phage therapy appears to be a promising therapeutic option. As demonstrated, phage therapy not only proved its value in PJIs but also in severe and hard-to-control infections of soft tissue, bone, and joints [65] or even systemic infections [66]. To date, phage therapy remains in the experimental stage, primarily due to the absence of large-scale clinical studies, particularly randomised controlled trials comparing two treatment arms: (1) phage therapy alone versus (2) phage therapy in combination with antibiotics. In Germany, approximately 55,000 revision surgeries of total knee and hip arthroplasties are performed annually [67], with numbers still on the rise. It is reasonable to assume that recruiting an appropriate study population would be feasible, given that approximately 20% of PJIs caused by *Staphylococcus aureus* result in treatment failure [68]. This raises the question of why there are still only a few clinical cases of PJIs treated with phages in the literature. The issue is certainly not a lack of available information, as numerous review articles have addressed this topic, either specifically focusing on phage therapy or mentioning it in the context of broader discussions on PJIs.

### Regulatory and clinical challenges

Not only according to our observations, but also as reflected in the current literature, it is primarily regulatory barriers that hinder the widespread clinical implementation of phage therapy. Therefore, we emphasise the urgent need for standardised clinical guidelines and therapeutic protocols. Ferry et al. [49] stated that, to implement phage therapy in healthcare systems, it is essential (1) to establish clear regulatory frameworks and quality standards for pharmaceutical-grade phages, (2) to build academic–industrial partnerships and national reference centers for production, testing, and clinical expertise, and (3) to identify relevant clinical indications and conduct controlled clinical trials to demonstrate efficacy and safety. To demonstrate the establishment of a national reference centre for phage therapy in France, Ferry et al. initiated the “PHAGEinLYON” program [53]. A corresponding initiative, “PHAGEFORCE,” was established in Belgium by Onsea et al. [69], proposing detailed methodologies to assess both the safety and efficacy of phage therapy in indications beyond PJIs. “PhagoDAIR” by Ferry et al. is a guideline for PJI exclusively [29], which could be taken as a blueprint for selected cases as stated above. To further validate their concepts, the authors registered what is, to the best of our knowledge, the only randomised controlled trial on this approach (ClinicalTrials.gov, NCT05369104), which is currently ongoing in Phase II.

We strongly believe that the implementation of phage therapy into clinical practice requires the establishment of specialized reference centers with multidisciplinary expertise in infectious diseases, microbiology, phage pharmacology, and surgical care. These centers would be responsible for phagogram testing, phage selection and manufacturing coordination, and therapeutic monitoring, while ensuring compliance with safety and regulatory standards. Due to the highly individualized nature of phage therapy, it is unlikely that this service could be effectively decentralized without compromising quality and safety. While exact numbers are not yet evidence-based, a minimal case load is required to maintain expertise, laboratory infrastructure, and clinical competence. In our opinion, based on analogy with reference centers for other rare infectious diseases, a reference center per 10 million inhabitants could be considered a reasonable starting point. This would allow for approximately 8 centers in countries the size of Germany, ensuring both accessibility and centralization of expertise.

### Treatment approaches and resistance considerations

The clinical application of phage therapy will continue to depend on the regulations set by local health authorities, particularly given its current status as a highly individualised treatment. In the development and clinical application of phage therapy, two principal strategies have been established: (1) the “prêt-à-porter” (off-the-shelf) approach and (2) the “sur-mesure” (tailor-made) approach, as described by Pirnay et al. [70]. The “prêt-à-porter” model involves the use of standardised phage cocktails composed of multiple phages with broad host-range coverage, making them readily available for rapid clinical use. In contrast, the “sur-mesure” approach involves the selection or isolation of patient-specific phages based on phagogram results, which is more time-consuming but potentially more targeted. Pirnay et al. [70] advocate for the “prêt-à-porter” strategy, emphasising the advantages of combining multiple phages over monophage therapy, particularly in the context of empirical treatment and in settings where rapid intervention is required. This preference gains further relevance in light of the high prevalence of polymicrobial and multidrug-resistant infections*—*an observation also reflected in our findings. The broader host coverage of phage cocktails increases the likelihood of effective bacterial targeting in such complex clinical scenarios. Furthermore, the use of multi-phage cocktails may help prevent the emergence of resistance, as suggested by Aslam et al. [37], who reported cases of phage resistance that were successfully managed by substituting the resistant phage with an alternative.

In the specific context of PJI, the clinical applicability of both strategies must be carefully weighed against the infection’s acuity, microbiological complexity, and surgical options. Acute PJIs, particularly those managed with implant-retaining procedures such as DAIR, often require rapid empirical therapy to prevent progression and preserve implant integrity. In such cases, the prêt-à-porter approach offers practical advantages: broad host-range phage cocktails are readily available and can be administered without delay, increasing the likelihood of early pathogen coverage, especially in polymicrobial or culture-negative infections. These off-the-shelf preparations also facilitate integration into standardized clinical protocols and may be more scalable within existing regulatory frameworks. Conversely, chronic or relapsing PJIs, where the pathogen is typically well-defined and embedded in a mature biofilm, may benefit more from the sur-mesure approach. Here, phagogram-guided (analogous to an antibiogram in antibiotic therapy) pre-sensitivity testing enables the selection of patient-specific phages for precise targeting of the persistent pathogen, including assessment of activity against biofilm-associated phenotypes. This strategy may be particularly useful when conventional antimicrobial options have been exhausted or are contraindicated. However, the logistical and temporal demands of producing personalized phage preparations, including regulatory approval and quality control, can pose a major barrier, as our own experience shows. Ultimately, in the treatment of PJI, a hybrid or adaptive strategy may be the most pragmatic solution: initiating empirical therapy with prêt-à-porter phage cocktails and adjusting treatment based on phagogram results and clinical response as individualized options become available*—*a concept already well integrated into the management of infections treated with conventional antibiotics.

### Clinical perspective and future directions

An advantage of phage therapy in the treatment of PJIs lies in the absence of pre-existing multi-resistance, as there is, to the best of our knowledge, no documented case of infection by bacteria that were not susceptible to any phage. This approach may also lead to a less invasive and more tolerable overall treatment regimen for the patient. A majority of reported cases employed a strategy incorporating implant retention (DAIR) or one-stage revision, thereby reducing surgical invasiveness by avoiding prosthesis removal. In cases where a two-stage revision would otherwise be indicated, this approach might therefore eliminate the need for another surgical intervention. However, all two- or multi-stage revision cases came out successful, but a robust evaluation in this regard is not feasible due to the limited number of available studies. Formation of a biofilm is ultimately the justifying indication for revision endoprosthetics in the case of infection. Studies indicate that phage therapy is effective against biofilms, at least when used in combination with conventional antimicrobial agents [23, 24].

## Limitations

While the reported outcomes of phage therapy in prosthetic joint infections appear promising, it is important to acknowledge the inherent limitations of the available evidence. The current body of literature is almost exclusively composed of case reports and smaller case series, which are subject to substantial publication bias. Positive outcomes are more likely to be published, whereas negative or inconclusive results may remain unpublished, leading to an overestimation of therapeutic efficacy. This bias limits the generalizability of the findings and underscores the need for prospective, controlled clinical trials to reliably assess the safety and effectiveness of phage therapy in this context.

It should also be noted that the literature search spans more than three decades. Over this period, substantial changes have occurred in surgical techniques, antimicrobial stewardship, and diagnostic standards, which may have influenced both the applied treatment protocols and the reported outcomes.

## Conclusion

This study highlights the potential of phage therapy for PJI. It provides a guideline for its administration, emphasising its high specificity, ability to target antibiotic-resistant bacteria, and capacity to disrupt biofilms. Despite encouraging outcomes, widespread clinical adoption remains limited by regulatory challenges, the lack of standardised protocols, and a lack of robust clinical trial data. Key prerequisites for clinical implementation include: (1) the establishment of clear regulatory frameworks and quality standards for pharmaceutical-grade phages, (2) the development of academic–industrial partnerships and national reference centres for phage production, testing, and clinical expertise, and (3) the identification of relevant clinical indications supported by controlled clinical trials to confirm efficacy and safety. Under these conditions, phage therapy represents a promising adjunct in the management of PJI.

## Supplementary Information

Supplementary Material 1.

## Data Availability

The datasets generated during and/or analysed during the current study are available throughout the manuscript.

## Abbreviations

CFU: Colony Forming Units
DAIR: Debridement, Antibiotics, and Implant Retention
PJI: Periprosthetic Joint Infection
