# Supplementary material for: Phage therapy in revision arthroplasty: State of the art and application protocols
Source: Arthroplasty. 2026 Jan 13;8:4. doi: 10.1186/s42836-025-00355-6 (PMC12797624; doi:10.1186/s42836-025-00355-6)
Supplement: Supplementary file 2 — Supplementary Material 1. [file 42836_2025_355_MOESM2_ESM.docx]

Using the building block approach, the following keywords/blocks were searched using the Boolean operator "AND/OR":

1. “phage therap*”[tw] OR “bacteriophage*”[tw] OR “phage*”[tw] OR “bacteriophage therap*”[tw] OR "Bacteriophages"[Mesh] OR "Phage Therapy"[Mesh]
2. "Arthroplast*"[tw] OR “joint replacement*”[tw] OR “THA”[tw] OR “TKA”[tw] OR “TSA”[tw] OR “endoprosthesis”[tw] OR “joint prosthesis implantation”[tw] OR “total hip arthroplasty”[tw] OR “total knee arthroplasty”[tw] OR “total shoulder arthroplasty”[tw] OR “total ankle arthroplasty”[tw] OR ”reverse shoulder arthroplasty”[tw] OR “unicondylar knee replacement”[tw] OR “hinged knee”[tw] OR"Arthroplasty"[Mesh] OR "Arthroplasty, Replacement"[Mesh]
3. "periprosthetic joint infection"[tw] OR “PJI”[tw] OR “arthroplasty infection”[tw] OR "prosthetic joint infection"[tw] OR "Prosthesis-Related Infections"[Mesh]
